# Drawing on the Arts to Enhance Salutogenic Coping With Health-Related Stress and Loss

**DOI:** 10.3389/fpsyg.2018.01612

**Published:** 2018-09-25

**Authors:** Ephrat Huss, Tali Samson

**Affiliations:** The Charlotte B. and Jack J. Spitzer Department of Social Work, Ben-Gurion University of the Negev, Beersheba, Israel

**Keywords:** oncological care, group work, salutogenic theory, positive psychology, art therapy

## Abstract

The connection between art therapy and specific theories of positive psychology such as Antonovsky’s theory of salutogenic sense of coherence (SOC) has been less articulated in the literature. This paper draws a methodological connection between art therapy and SOC, that is, meaning, manageability and comprehensibility, as the components of coping. This theoretical and methodological connection is then explored with a group of participants dealing with the health-stress of cancer.

**Method:** We conducted a large-scale, qualitative study that included fifty transcribed hours of thematically analyzed arts processes and one hundred art works, used to explore salutogenic theory within a support group for recovering oncological patients.

**Results:** The results point to the arts as including mechanisms that enhance meaning, manageability, and comprehensibility in an embodied and synergetic way. The art makes it possible both to separate and to ‘fill’ these three components, while on the other hand, integrating them into a cyclical element. We outline theoretical and methodological implications of understanding art therapy as a methodology to enact and concretize positive psychology theories, as well as presenting a protocol for using arts to enhance salutogenic coping in the context of health-related stress.

## Introduction and Literature Survey

Salutogenic theory, as part of positive psychology, has proposed the novel concept of ‘salutogenesis’ (the origin of health) for research into stress, as an approach that has contrasted with the concept of pathogenesis or the study of stress pathology and disease development focusing on trauma. The salutogenic model proposes that the goal of health research should be to identify, define, and describe pathways, factors, and causes of positive health to supplement our knowledge about how to prevent, treat, and manage negative health (pathogenesis) (Antonovsky, 1979). Thus, the state of emotional and physical health of each individual is an interactive spectrum between stress and coping (Antonovsky, 1979). Antonovsky broke down the concept of coping into the ability to conceptualize the world as manageable, understandable, and meaningful. These together create a sense of coherence (SOC) which plays an important role in the way one perceives challenges throughout life. SOC is an enduring tendency to see the world as more or less *comprehensible* (the internal and the external world are perceived as rational, understandable, consistent, and expected), *manageable* (the individual believes that s/he has the resources needed to deal with situations), and *meaningful* (the motivation to cope and a commitment to emotionally invest in the coping process are present) (Antonovsky, 1979). Given their tendency to perceive the world as comprehensible, meaningful, and manageable, individuals with a strong SOC will be less likely to feel threatened by stressful events and will be better equipped to adjust to them (Eriksson and Lindstrom, 2005; Eriksson et al., 2007).

Interestingly, Antonovsky also claimed that salutogenesis can be intensified and consolidated over a lifetime, that is, thinking about and defining SOC can be a way to create SOC. However, he did not elaborate on how this could be done. Numerous studies recently published have shown that SOC may be considered a protective factor during adolescence and that it may contribute to moderating and mediating stress experiences (e.g., Moksnes et al., 2011, 2012; Garcia-Moya et al., 2013a,b).

However, SOC is used more as a research tool, and its concepts are abstract and intellectual when working with different cultures. The arts may be a way to embody, concretize, and internalize the elements of SOC, namely meaningfulness, manageability and comprehensibility, and thus, to enhance them. Conversely, this theory provides a more detailed framework of concepts for art therapy to focus on coping rather than on pathology, and thus, to become a relevant methodology within positive psychology. A more complex theoretical and methodological basis to integrate arts into positive psychology, or to use positive psychology within art therapy, is just emerging and needs to be connected to specific positive psychology theories in specific ways (Huss, 2015).

### Art Therapy as Embodying Sense of Coherence

Salutogenic theory contains the basis of behaviorist therapy by focusing on management, but it also contains conceptual and existentialist-humanistic elements by focusing on comprehension and manageability (Carstens and Spangenberg, 1997; Amirkhan and Greaves, 2003; Luutonen et al., 2011). Focusing attention on these three elements – manageability, comprehension, and meaning – and situating these elements within specific situations and narratives may be a way of intensifying them. Art therapy can be a suitable method for enhancing embodied management elements; manageability as a type of embodied aesthetics can thus include elements beyond abstract conceptualizations, as in verbal therapy (Mohanty, 2003; Huss, 2007, 2010, 2012). Art also enhances meaning making and comprehensions by enabling these three elements to interact in the process, product, and explanation of art work. More specifically, creating arts enables enhanced stress management, but at the same time, makes it possible to reflect on the content, understanding it and reaching more enabling levels of meaning so as to create an overall SOC. In other words, the arts include a component of ‘doing’ that, in effect, is parallel to the management element. To elaborate, using art as a main methodology to express SOC components becomes an action-based activity in which the participant ‘manages’ decisions about what and how to depict on the page; figures, images, colors, materials; and more are all managed by the artist to create a narrative. Indeed, Antonovsky (1979) recommended that the salutogenic model might be applied in action research.

In terms of comprehensibility, the literature on multi-literacy learning points to the arts as providing a concrete and spatial interactive gestalt that can incorporate multiple elements of coping, as compared to the more linear verbal analyses. This enables valuing pictures of coping that have internal compositional coherence in a complex gestalt that incorporates both the stressor, but also the context of the stress, in the relationship between figure and background. This facilitates new, integrative solutions that can include moving closer or farther away, merging, separating, or changing the size and contours of shapes, and centralizing or decentralizing the overall gestalts of the system as a way to manage them (Huss and Cwikel, 2008; Huss, 2012). This helps to reframe meaning, as well.

Overall, the arts as an embodied language are cited as involving a complex dialog between emotion, cognition, and the senses or body that prompts fast, perceptual processing and information gathering while inducing metabolic arousal that mobilizes the organism for managing stress (Nelson and Fivush, 2004; Hass-Cohen and Carr, 2008). The visual gestalt enables a broad, hermeneutic base to reach new meaning structures. Indeed, people have always used the arts to address and express pain and adversity, so as to enhance their resilience through embodied symbolic interaction and self-expression (Kaye and Bleep, 1997; Zelizer, 2003). The arts, as stated above, recreate a connection between cognition, emotion, and the senses (Csikszentmihalyi, 1990). Arts provide an accessible source for the retrieval and interpretation and reinterpretation of stressful sensory experiences, such as illness and medical interventions in the ASD period when the sensory stress image is still flexible within memory (Nelson and Fivush, 2004; Huss and Sarid, 2012).

This salutogenic conceptualization of art therapy differs from the dynamic understanding of art as a projective expression of the unconscious. It is also different from fine art that is focused on the product rather than the process. Art, as described above, including process, product, and interpretation, becomes an embodied aesthetic experience but also a broad phenomenological space for embodying and concretizing a person’s meaning, manageability, and comprehensibility in a single ‘coherent’ art work (Huss et al., 2017).

After arguing a theoretical case for the potential of arts to create a methodology for enhancing SOC for salutogenic coping, we wish to present evidence of how this works in a support group for oncological patients with the stress of treatment and recurring cancer. This fits Antonovsky’s focus on health-related stress. Dealing with cancer is a long-term coping challenge that includes both acute and long-term stress components. This research questions how the arts can be used to concretize and embody, and thus enhance, salutogenic coping.

### Salutogenic Coping, Art Therapy, and Oncological Care

Cancer is a life-threatening disease that arouses great stress, although survival is increasing thanks to advances in medicine and technology. Cancer survivors are under constant stress, suffering many types of loss and worrying about whether the illness will return. There are three central stages in dealing with cancer: the diagnosis, the treatment, and the long-term survival. Problems include fear of dying, coping with the medication, extreme anxiety issues with body image, sexual malfunction, and family problems to name a few. The connection between stress and coping with cancer has been documented in the literature. Thus, learning to cope with stress can help manage the cancer outcomes. The methods of working with the stress of cancer are diverse, and there is no single method proven most effective (Wilkinson and Kitzinger, 2000; Pérez et al., 2014).

Art therapy and oncological care can be divided into literature on art therapy as psychological intervention, and into arts in health as a more macro-oriented general orientation. Art therapy literature describes the use of arts for dealing with cancer as the space to non-verbally describe feelings and thoughts indirectly that are too anxiety provoking. Using images and metaphors can help express this fear-inducing content indirectly. This, on a systemic level, enables communication within the family to occur in the safe and distanced zone of the arts (Wood et al., 2011).

Another conception of art therapy within cancer is the use of art to ‘humanize’ the patient as a creative person who also has cancer. The arts provide the patient with space to express him or herself beyond the illness level as a whole person. In the context of the dehumanizing and invasive experience of hospitalization, the arts can provide a space where the patient is in control, can make decisions, and can self-regulate the amount of disclosure (Minar, 1999; Balloqui, 2005).

Arts in health literature also describes the arts as helping to humanize the hospital and to create a more welcoming and flexible environment that influences the doctors, nurses, families, and patients on a macro-level. Aesthetic pleasure helps to counteract the painful interventions and aspects of the illness. Arts are also used as a way to distract from the pain of the illness and from worries and over-thinking about the illness, such as in medical clowning (Malchiodi, 1999; Gilboa-Negari et al., 2017).

Based on the above literature, our assumption is that the arts process has inherent salutogenic elements connected to the levels of art materials, compositional elements, and discussion of the art product that embody and enhance management, meaning, and comprehensibility. This paper aims to further explore how art methods help enhance salutogenic coping with a group of recovering cancer patients.

## Materials and Methods

### Research Strategy

Because this research was preliminary and explorative, hoping to create nuanced connections between arts and SOC, we utilized qualitative evaluation to understand the interaction between arts and the salutogenic theory within support groups. Our aim was to create and to validate theoretical and methodological connections between arts and SOC. Future research, based on the findings of this paper, will be used to validate these findings quantitatively and to expand them to additional population groups.

#### Field of Research

This research took place in a support group at a support center in Israel for people and their families dealing with and recovering from cancer. The framework provides community psychological and psychosocial support activities for those recovering from cancer, enabling them to build a supportive social group and set of activities to replace work, such as arts and lectures, as well as social services and therapy. Most participants are between the ages of 30–60, were all female, except one, and were at different stages of their illness and of potential recovery but have not yet returned to work. Some were jut experiencing remission of the illness. The demographics of the groups are thus broad and changing as it is a train group where some leave (due to illness, death, or recovery and some stay for a long time). Because of this, the groups cannot be compared, and there were constant fluxes and differences between the groups- although the methods of using the arts were replicated. In this context, we offered a 12-meeting social support group using salutogenic theory through the arts.

#### Protocol of Salutogenic Art Therapy Meetings

At each meeting, we presented an art warm-up, such as choosing a color symbol or using art material to express ‘how I feel’ today. We then asked participants to present something that was causing stress that day through art and provided subject structures for those who wanted them, such as my family or images of cancer or an upcoming event that scares me or a current dilemma. After creating the stress image, the drawer explained the image to the group, and the group helped to think of meaning, manageability, and comprehensibility components that could help to cope with the stressor. The drawer could then change their image if s/he wished, to include these coping elements.

#### Ethical Considerations

Researchers explained to the participants that the group was a research group exploring the usefulness of salutogenic theory through arts, and members could participate without agreeing to be part of the research (that is, all of their texts and drawings would not be used in the research). All elements were kept anonymous. We received clearance from the Helsinki hospital ethics committee for this research from Soroka Hospital, as the self-help group is connected to this hospital.

#### Data Sources

We transcribed the recorded, verbal interaction and photographed the art works of two groups of 10 participants each that went on for 12 weeks, each group meeting for an hour and a half. This rendered 50 h of transcribed group work time and over 100 pictures as data sources. Additional data included ten semi-structured, retrospective interviews with participants from the groups so as to access phenomenological understanding of how they experienced the arts and how the salutogenic theory was interconnected.

### Analytical Strategy

#### First Analysis

The interactive elements of creating an art product, explaining it, and sharing it with the group, and defining the meaning, manageability, and comprehensibility components were first analyzed narratively as an ecological whole. This meant that the analysis followed a narrative framework, moving from:

(1) The creator’s phenomenological explanation of the art work,
(2) To the interactive process of responding to the image by the group, and
(3) To additional thoughts of the authors when overseeing this (Huss, 2015).

#### Second Analysis

All of these levels were divided in terms of salutogenic SOC components. These elements of meaning, manageability, and comprehensibility in art use were clustered into overall themes.

### Validity and Reliability

The triangulation of data sources, including verbal, visual, narrative, and semi- structured, and the repetition of all of the data collecting twice, worked to enhance reliability. The peer expertise of the two authors, one an expert in cancer stress and the other an expert in arts therapy, also helped to validate the paper (Morse, 1995). Additionally, the group was a shared reality group that responded to the images, and thus helped to validate the contents as in participatory research methods (Denzin and Lincoln, 1994).

## Results

### Data Presentation and Discussion

As stated in the methods section, in order to share the first narrative element of the meetings as an interaction between the art process, product, and discussion occurring over time in the group space, we have chosen a few complete examples of specific images and interactions as illustrations.

#### First Analysis: Holistic Analyses of Five Examples

The following examples were chosen because they exemplify five different issues in dealing with cancer (O’Connor et al., 2011; Kwak et al., 2013). They also exemplify different connections and interactions between meaning, manageability, and comprehensibility as expressed through the art process and product.

##### Example No. 1: Learning to live with cancer in the long-term

**Figure:**
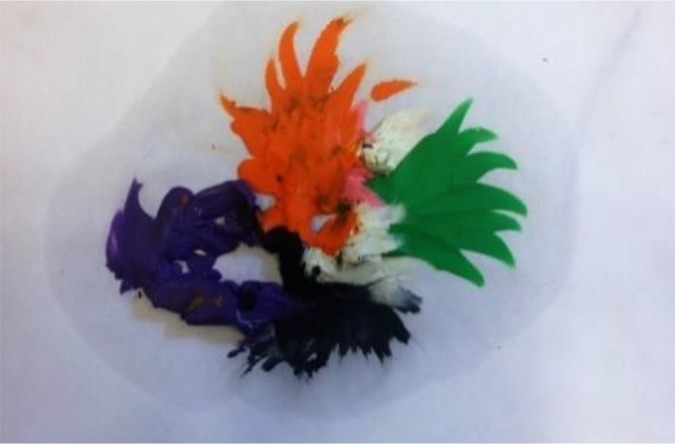

*Art process as manageability.* (Narrative to group) “I chose clay because it enabled me to ‘do’ something, and the material is malleable and can be transformed into something else. It’s important for me to ‘do’ something in order to feel that I can manage the situation. Kneading the dough is calming and enabled me to manage my emotions concerning cancer.”

*Meaning level from observing the image and explaining it to the group.* “While placing the clay on the page, I was in constant dialog with myself about the amount of space that each part should receive and how the parts should be connected. It was important for me to put the most meaningful elements, family and work, at the top, and the cancer at the bottom.”

“The black clay represents illness and death. The other colors represent other elements of life such as family (orange), career (green), religious and spiritual elements, my synagogue (white), and my social life, leisure activities, and others (purple).”

“The cancer is black, and it’s at the basis of everything. In effect, the black clay is under the other colors. It’s at the basis of everything, but it’s in control because of my ability to utilize my spiritual beliefs.”

“I think that this image shows how we have to understand that we can’t separate the presence of cancer from our lives; it doesn’t work. The black color is always there at the base, and so I am going to move it upward a little so that I remember it’s role, and I don’t try to hide it again. I’m also going to enlarge the purple, which is self –care, and includes for me, sports, and meditation- and I will put the orange, the family, that is most important, in the middle…”

We see in this process of choosing materials the ability of art to create an embodied experience of manageability in the here and now of the art process. The experience of manageability is in the here and now, as compared to the lack of manageability of the illness. This embodied manageability enabled participants to overcome the ‘freeze’ reaction in stress situations. The description of the sensory interaction with the clay leads to cognitive flashes of understanding in terms of what each color represents. This is enabled in the relative and whole gestalt, and in turn, creates insights that are emotion-led and become meaningful in terms of the hierarchy and placement of the colors. Finally, this leads back to shifts in action – in management of the colors – as their placement is changed, based on these new meanings. This process is thus spiral, developing, and involves managing a complex gestalt of different elements that become hierarchized and imbued with meaning.

In the shared-reality group, this became a type of salutogenic coping on all three levels, producing an example of how to manage cancer, namely, the ability to hold both fear of illness and health parts together. It also defined what gives strength, that is, activating spiritual beliefs and leisure activities so as to cope with the cancer as a long-term entity and to define what is most important. These outcomes are based on participant rather than on expert knowledge. The need to explain this art, rather than only to observe it or to let someone else explain it, created an intensification and connection between the three elements at the basis of the concept of SOC, specifically, the overall gestalt of art creating, observing, and explaining together with the compositional decisions taken in creating the art interaction to create coherence. The understood importance of activating self-care because of the comprehension is that the cancer could not be taken out of the image. This became a reason to find ways to manage it, so as to give attention to the more meaningful elements, such as the family. This example was manageability-driven with the manageability calling for a shift in understandings and meanings that was then able to impacted it.

##### Example No. 2: The challenge of understanding medical procedures

**Figure:**
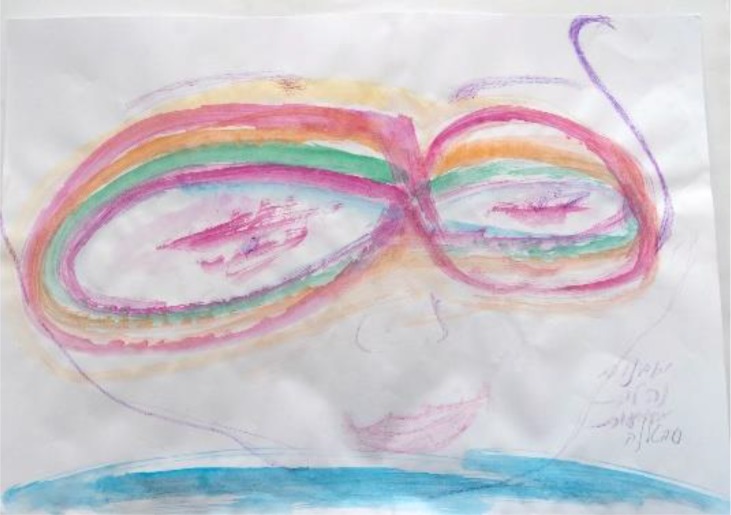

Artist: “This is my art work: 3 years have passed since this event, that I now understand was a huge trauma – drawing this picture, I understood that the biggest trauma was having to do special chemo treatments in Jerusalem. I became asthmatic in the middle [of the treatment], and it turned out it was the wrong machine for me. And it was such an effort to reach Jerusalem when I was so sick. I understood while I was on the machine that something was wrong and then I was told that it [the medication] had hurt my heart. I felt such helplessness and anger at myself that I hadn’t checked it out but instead trusted the doctors. I drew this page of a person whose eyes are closed (cries).”

Group Member 1: “But you did tell them that you felt something was wrong, you did what you could…it could have been worse. You did what you could facing a large system like a hospital, and you managed to stop the treatment. Maybe you made the damage to your heart less. You did manage it.”

Group Member 3: “And the meaning doesn’t have to be that you are helpless, or that you should be angry at yourself but that you did the best you could. We all do the best we can; we don’t fully understand the illness, neither do the doctors…”

Artist after this conversation, taking colors and adding glasses to the drawing: “I have changed my image from unclear eyes to glasses that show that I was trying my best to understand, and I did stop the medication. I did my best.”

In this example, we see that the subject of comprehension, that is, understanding the medical treatments, is central. The lack of comprehension led to an experience of lack of management. The missing component of new meaning (‘I did the best I could’) was provided by the group’s encouragement and shared-reality understanding. The artist actively changed (or managed) her image through this new meaning, after shifting the comprehension of the situation. The meaning shift here was dominant, leading to new comprehensions and a sense of manageability that together created SOC.

##### Example No. 4: Finding meaning in having cancer

**Figure:**
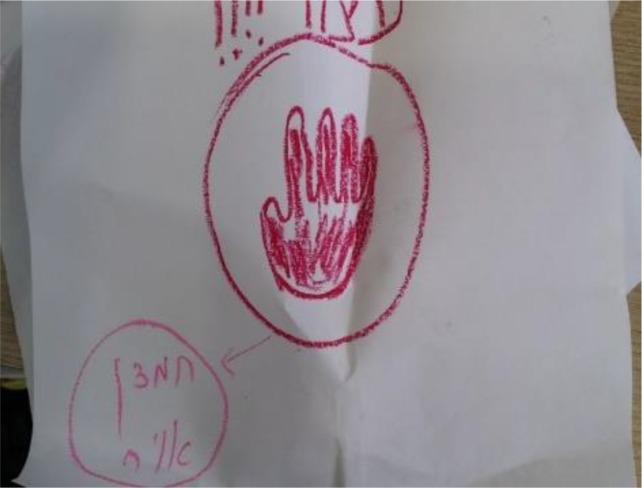

Artist: “The meaning of my life was always to do everything for everyone in my family, to manage everything. I was the center, that was the meaning of being a good wife and mother. Not anymore, not since the illness. Now I know that I have to put myself first. I manage myself in a new way, I put myself first always and say, ‘Stop.’ I rest, I don’t get involved in everyone’s problems. I look after myself. Now, I manage myself differently, and this gave me new meaning to my life and to my relationship with myself. Everyone has learned to manage – everyone manages fine, maybe even better. I have understood that I am responsible for myself, that I am important to myself. I want to thank the illness for that, so I drew a red stop sign. After looking at this image, I went over the red stop sign again, in a stronger color, so as to make my message very clear to others and to myself.”

In this example, the woman describes an intense shift in comprehensibility (that she has to put herself before her family) that led to intense shifts in manageability (behaving differently in her family) and that led to finding positive meaning in her illness. In other words, the new comprehensions created new behavior and created new meaning. This is all in the explanation process and symbolized by the stop sign that she drew. The art explaining is thus more central than the art process, although the act of drawing the stop sign can be seen as an active way of managing her behavior in the family.

##### Example No. 5: Denial of the illness

Artist: “I drew a picture of a river; everything is calm, everything is ok. I feel ok, thank God, and I drew pebbles by the river, that I now – looking at the picture – understand that they are my sisters. They helped me so much. My husband, he couldn’t stand seeing my pain. I know he loves me, but it was harder for me, and he doesn’t know how to look after the house, but they came, they cooked, they helped me.”

Participant: “What is that black cloud in the right-hand corner?”

Artist: “I don’t know. I think, now that you asked, that that is the test result I am waiting for. There is one test result that I’m very stressed about, and I’m waiting.”

In this excerpt, we see how the compositional questions of the group member (‘What is that cloud?’) helped to overcome denial and create comprehension of the denial itself. Through comprehending the fear of the test, the denial level of management was shifted to acknowledgment and verbalization of fear. This comprehension was then transferred to the husband’s denial of the illness. Thus, explaining the art work in the group helped overcome the management style of denial.

#### Second Analysis: A Typology of Methods for Enhancing Sense of Coherence Through Art Creation, Observation, and Discussion

The above examples holistically and narratively show how art can enhance and connect between meaning, manageability, and comprehensibility in different ways. From these, and the additional 60 art examples, a set of central strategies for using art to enhance meaning, manageability, and comprehensibility are presented in the following lists.

##### Methods of creating manageability in the art-making process and explanation

(a) Managing the art materials (‘I kneaded the clay until it was soft enough to work with…’).
(b) Managing the compositional organization of the image (‘I started by placing the black part at the bottom’).
(c) Regulating disclosure of content when explaining the image (not talking about elements, crying when explaining emotional elements).
(d) Managing shifts in comprehension and meaning that lead to shifts in manageability in the here and now of the art process or explanation.

##### Methods of creating comprehension in the art-making process and explanation

(a) Defining and filling in the gaps between the overt content level and the visual depiction metaphors, symbols, metonyms, and compositional elements such as size and shape (‘I didn’t know where to put the black, and then I understood I didn’t want the black to be anywhere – I was not accepting the illness,’ i.e., ‘I understood the importance of × so I moved this shape to the center’).
(b) Defining and filling in gaps between content and composition in the art observation (‘I understood that the stones by the river are my sisters’).
(c) Defining and filling in gaps through others’ observations and questions (for example, when participants asked what the cloud in the corner was and it caused the drawer to face their fear of the test results).
(d) By explaining one’s image and using compositional language, new ‘knowledge’ about the image can be excavated by the artist (‘What is the purple shape?’ ‘It is self-care; it includes spirituality and sports for me’).
(e) Others providing new explanations (for example, a participant saw the black shadow that the drawer did not ‘see,’ assumed it had meaning, and demanded an explanation for it).
(f) Interrelationship between different elements in the spatial gestalt of art (for example, adjusting the overall placement of elements in terms of which is largest, which is most central…).

##### Methods of creating meaning in the art-making activity

The comprehension and meaning levels of the interaction with the art work are often overlapping, but meaning emerges from the intuitive actions that are observed, and hold a more emotion-laden content:

(a) Reflecting on intuitive actions and decisions in the art-making process to understand the emotional content (‘I felt that the orange was the most important part. It was important for me to strengthen the red around the stop sign’).
(b) Exploring the overall meaning levels in the context of the whole art gestalt (‘While my husband is drawn at the side, my sisters are close to me. But he loves me, he just can’t handle the illness’).
(c) This is the integration of the whole gestalt: what is figure, what is background, as well as integration of emotion and cognition through intuitively finding central and peripheral elements in the image and narrative.
(d) The combination of excavating a narrative in an embodied, but also relational, way enabled meaning to be created, if we define meaning as a meeting place between emotion and cognition, or subjectivity and reality.

##### Art as enhancing overall SOC through integrating the three components

(a) An interactive process between managing the art materials, understanding the content, and providing new meanings.
(b) A process that can spirally repeat all of these stages in different orders (for example, adjusting the art work after the discussion or understanding something new while creating it). This can include both physical adjustment, or adjustment on the level of new understandings of the same image, or adjustment in terms of experiencing new meaning from the image, or all of these.
(c) Showing which elements are lacking (for instance, there may be meaning but this is not translated to manageability or vice versa).

## Discussion

From the above narrative and content analyses of the data, we reached a typology of art to enhance salutogenic coping in stress situations such as in dealing with cancer. These will be discussed in relation to literature on the salutogenic theory, on coping with cancer, and on arts therapy.

We saw that the art work raised typical stressors of dealing with cancer, such as learning to live with the cancer in the long term, the challenge of understanding medical procedures, finding meaning in having cancer, and issues of denial. These were translated into narrative form through examples of specific events or situations, and into metaphorical form through symbols of different elements. The art enabled entry into the subject by how it was managed, understood, or by the meanings attributed to it. The results showed the cancer as both a traumatic event, but also, as an event that enabled a new SOC to emerge, that is, enabling posttraumatic growth. The interesting thing about the art was that it allowed for showing both the stress situation and the ways it was coped with, or the growth, simultaneously as interactive parts. Because the stressor was concretely portrayed, the ways it generated SOC could be applied directly to that stressor.

In terms of methodologies of creating meaning, manageability, and comprehensibility out of the cancer experience, we saw that manageability was created in the art-making process and explanation was achieved in managing the art materials (‘I kneaded the clay until it was soft enough to work with’), managing the compositional organization of the image (‘I started by placing the black part at the bottom’), regulating disclosure of content when explaining the image (not talking about elements, crying when explaining emotional elements), as well as managing shifts in comprehension and meaning that lead to shifts in manageability in the here and now of the art process or explanation.

We see above that the act of actively creating and interpreting art (rather than being interpreted or diagnosed by an expert) created a symbolic but also an embodied type of experience of interaction with and management of the ‘world’ through the orchestration of art materials, meanings, and compositional depictions of those meanings. This connects to theories of embodied aesthetics that go beyond the passive ‘looking’ and the perceptual process to include multiple components such as sensory stimuli, smell, texture, color, and movement of the art materials and of the body that create a multisensory embodied aesthetic experience (Shapiro, 2014). This embodied experience of art materials connects to percpetual and cognative elements of autobiographical memory that create a web of cultural and personal associations around the sensory experience, enabling new understandings and meanings of the art making process while creating art (Nelson and Fivush, 2004).

Art also enables an additional type of management in that it enables tactile expression of feelings that helps to sublimate these feelings (such as pounding clay to make it softer above). Art materials come in a range of fluidity and intensity of color that can express the need for control by using pencils, or alternatively, the need for letting go by using more fluid elements such as water colors. Art materials can be contained or ‘messy,’ and the creator can choose materials that regulate his emotional needs at the time (Rubin, 2001). The use of colors and shapes enables one to access, and thus manage, difficult emotions as shown by the participant who did not notice the cloud they had drawn until looking back on their art work. This distances intense emotions onto the page so that one can ‘observe’ the content as outside of the self. The use of symbols and metaphors also helps to regulate emotional reactions, including self-regulation and management of difficult situations. The final stage of actively changing the art work also creates a symbolic zone of management (for example, putting glasses on the eyes so as to observe medical procedures more carefully, as in example 2) (Huss, 2012, 2015).

Methods of creating comprehension in the art-making process and explanation included defining and filling in the gaps between the overt content level and the visual depiction. It also included defining and filling in gaps between content and composition in the art observation and defining and filling in gaps through others’ observations and questions. By explaining one’s image using compositional language, new ‘knowledge’ about the image can be excavated by the artist. This also occurred with others, providing new explanations. This can be explained through the mechanisms of art-making that involves an intense dialog between form and content, helping to more exactly define and redefine the content level as understood by the artist, and not by an external system, power holder, or expert. This gives voice, space, and legitimacy to all of the possible interpretations of the issue (Huss, 2015; Hafford-Letchfield and Huss, 2018). Creative problem-solving theories show how the shift to a visual language shakes the system of thinking, and thus, enables new ‘visual’ perspectives of the problem, in terms of its overall gestalt. This process continues into observing art. Betinsky, a phenomenological art therapist describes how engaging the client in observing ‘what we see’ in terms of compositional elements enables production of new explanations and associations (Betinsky, 1995; Beraby-Meyer et al., 2004).

Another element of discussing art within a group of shared reality is that it provides a range of experience of others in the same situation. This helps to situate the experience within a social context, defining the context as the problem, rather than the participant. We could see that others had also experienced difficulty understanding medical decisions, that others also understood the need to put self before family, and to accept that some family members would not be able to be supportive. This shared reality makes it possible to situate the stressors within their social context, as in critical theories, also by defining what the ‘figure’ is and what the background is (Kapitan, 2003; Huss, 2015).

Finally, methods of creating meaning in the art-making activity, including the comprehension and meaning levels of the interaction with the artwork, are often overlapping. But meaning emerges from the intuitive actions that are observed and that hold more emotion-laden content, reflecting intuitive actions and decisions in the art-making process in order to understand the emotional content, as well as exploring the overall meaning levels in the context of the whole art gestalt. Similarly, meaning in the discussion included the combination of excavating a narrative in an embodied, but also in a relational, way enabling meaning to be created, if we define meaning as a meeting place between emotion and cognition or subjectivity and reality. Indeed, art making and observing has been defined neurologically as an integrative activity that integrates left and right brain functions, and as such, creates new neurological pathways between emotional and cognitive areas of the brain, enabling flexibility of thought, as against the rigid, repetitive, or fragmented thinking when under stress or after trauma (Csikszentmihalyi, 1990; Hass-Cohen, 2003).

Finally, this meaning, manageability and comprehension were adjusted and integrated to create the overall SOC. While Antonovsky discussed SOC, he did not explain in detail how these three elements interact. From this art intervention, we see that what is important is the integrative element of meaning, manageability, and comprehensibility into a complex, cyclical interactive process. This includes separating and defining each element of the salutogenic SOC. If one is missing, then it can be ‘filled in’ in the art-making or explaining process. This synchronicity between comprehensibility, meaning, and manageability is what creates ‘coherence’ in Antonovsky’s terms. It is achieved on an embodied, reflective, and also interactive level. The group level was also seen to activate a collective SOC emerging from a shared reality (Liebman, 2003). This may be the biggest advantage in using art to enhance salutogenic coping. The protocol of art-making enables granting time at the end of the art-making to adjust one’s image if this is wanted, based on the sharing experience (Riley and Malchiodi, 1994). We have seen that some participants adjusted their images, initiating a second spiral of management, meaning, and comprehension.

In this cycle, the image is ‘re-managed’ and ‘re-imagined,’ based on the comprehensions and meanings created. Thus, in the first example, a participant made more room for and enlarged the black ‘cancer’ shape after understanding that it could not be hidden. She also put the family at the center, as the most meaningful element for her. This second cycle can create a whole new set of meanings, comprehensions, and manageability. This may include both physical adjustment, or adjustment on the level of new understandings of the same images, or in terms of experiencing new meaning from the image, or all of these. This enables ‘filling in’ the element of SOC that is missing or that is weakest, so as to create an overall gestalt of solutions of the ‘mind’ that is comprehension, the ‘heart’ that is emotion, and the ‘doing’ that is manageability. This integrative effect is different from cognitive processing alone as in CBT, from existential- and meaning-oriented interventions and from behavioral or ‘doing’ interventions. The strength of art is thus similar to the strength of the concept of SOC in that it demands an integration of all of these elements.

A limitation of this study is it’s descriptive and theoretical nature: that it did not measure salutogenic coping before and after the intervention or compare it to a control group to see how art impacts salutogenic coping on an empirical level. This does not enable exact replication of the study. This leads to another limitation- that we did not include quantitative methods to validate this model. Future research can go in these directions. An advantage is that it created a rational for the connection between art and salutogenic theory. In other word, its strengths are connected to this limitation in that it enabled to carefully explore the methodological, and theoretical connections between the theory and the art use, through highlighting the specific mechanisms of art that connect to the salutogenic theory. Its strengths are providing a theory- based protocol for connecting between arts and salutogenic coping. Future research can evaluate and validate the directions outlined in this qualitative study.

This has intense implications for theoretical and methodological connections between art therapy and positive psychology, using theories of embodied relational and phenomenological aesthetics as mediating elements.

The data above showed how the arts can be used to enhance, embody, and develop the three elements of salutogenic coping – meaning, manageability, and comprehension. On the level of salutogenic theory, we learned how the arts help to concretize the framework of salutogenic theory that integrates solutions of the body or legs’ doing, the “heart” and “brain,” creating an embodied perceptual, emotional, and relational integration between different ways of coping. It also provided a clear methodology that is based on theory, rather than on art ‘recipes’ or general, romantic proclamations about art being healing.

## Author Contributions

EH is the first primary author, wrote first draft and initial analyses. TS is the second author, helped with analyses and editing and the conceptualization.

## Conflict of Interest Statement

The authors declare that the research was conducted in the absence of any commercial or financial relationships that could be construed as a potential conflict of interest.

## References

[B1] Amirkhan J. H. Greaves H. (2003). Sense of coherence and stress: the mechanics of a healthy disposition. *Psychol. Health* 18 31–62. 10.1080/0887044021000044233

[B2] Antonovsky A. (1979). *Health, Stress, and Coping: New Perspectives on Mental and Physical Well-being.* San Francisco, CA: Jossey-Bass Therapy, 39.

[B3] Balloqui J. (2005). “The efficacy of a single session,” in *Art Therapy and Cancer Care*, eds Waller D. Sibbett C. (Maidenhead: Open University Press), 128–136.

[B4] Beraby-Meyer Y. Moran S. Unger-Aviram E. (2004). When performance goals deter performance: transfer of skills in integrative negotiations. *Abstract Organ. Behav. Hum. Dec. Process.* 93 142–154. 10.1016/j.obhdp.2003.11.001

[B5] Betinsky M. (1995). *What Do You See? Phenomenology of Therapeutic Art Experience.* London: Jessica Kingsley.

[B6] Carstens J. A. Spangenberg J. J. (1997). Major depression: a breakdown in sense of coherence? *Psychol. Rep.* 80 1211–1220. 10.2466/pr0.1997.80.3c.1211 9246887

[B7] Csikszentmihalyi M. (1990). *Flow: The Psychology of Optimal Experience.* New York: Harper Collins.

[B8] Denzin N. K. Lincoln Y. S. (1994). *Handbook of Qualitative Research.* Thousand Oaks, CA: Sage Publications, Inc.

[B9] Eriksson M. Lindstrom B. (2005). Validity of Antonovsky’s sense of coherence scale: a systematic review. *J. Epidemiol. Commun. Health* 59 460–466. 10.1136/jech.2003.018085 PMC1757043 15911640

[B10] Eriksson M. Lindstrom B. Lilja J. (2007). A sense of coherence and health. Salutogenesis in a societal context: aland, a special case? *J Epidemiol. Commun. Health* 61 684–688. 10.1136/jech.2006.047498 PMC2652992 17630366

[B11] Garcia-Moya I. Moreno C. Jimenez-Iglesias A. (2013a). Understanding the joint effects of family and other developmental contexts on the sense of coherence (SOC): a person-focused analysis using the Classification Tree. *J. Adolesc.* 36 913–923. 10.1016/j.adolescence.2013.07.007 24011107

[B12] Garcia-Moya I. Rivera F. Moreno C. (2013b). School context and health in adolescents: the role of sense of coherence. *Scand. J. Psychol.* 54 243–249. 10.1111/sjop.12041 23418864

[B13] Gilboa-Negari Z. Abu-Kaf S. Huss E. Hain G. Moser A. (2017). The cross- cultural perspective of medical clowning: comparison of its effectiveness in reducing pain and anxiety among hospitalized Bedouin and Jewish Israeli children. *J Pain Res.* 10 1545–1552. 10.2147/JPR.S135678 28740420 PMC5505546

[B50] Hafford-Letchfield T. Huss E. (2018). Putting you in the picture: the use of visual imagery in social work supervision. *Eur. J. Soc. Work* 21 441–453. 10.1080/13691457.2018.1423546

[B14] Hass-Cohen N. (2003). Art therapy mind body approaches. *ProgressFam. Syst. Res. Ther.* 12 24–38.

[B15] Hass-Cohen N. Carr R. (2008). *Art Therapy and Clinical Neuro-science.* London: Jessica Kinsley.

[B16] Huss E. (2007). Symbolic spaces: marginalized Bedouin women’s art as self- expression. *J. Hum. Psychol.* 47 306–319. 10.1177/0022167807301891

[B17] Huss E. (2010). “Bedouin women’s embroidery as female empowerment,” in *Materials and Media in Art Therapy*, ed. Moon C. (London: Routledge), 130–145.

[B18] Huss E. (2012). Integrating strengths and stressors through combining dynamic phenomenological and social perspectives into art evaluations. *Arts Psychother.* 39 451–455. 10.1016/j.aip.2012.07.001

[B19] Huss E. (2015). *A Theory-based Approach to Art Therapy: Implications for Teaching, Research, and Practice.* London: Routledge.

[B20] Huss E. Bar-Yosef K. Zaccai M. (2017). The meaning of flowers: a cultural and perceptual exploration of ornamental flowers. *Open Psychol. J.* 10 140–153. 10.2174/1874350101710010140

[B21] Huss E. Cwikel J. (2008). Embodied drawings as expressions of distress among impoverished single Bedouin mothers. *Arch. Womens Mental Health* 11 137–147. 10.1007/s00737-008-0007-8 18493707

[B22] Huss E. Sarid O. (2012). “Using imagery to address physical and psychological trauma,” in *Art therapy and Healthcare*, ed. Malchiodi C. A. (New York, NY: Guilford Publications), 136–145.

[B24] Kapitan L. (2003). *Re-enchanting Art Therapy: Transformational Practices for Restoring Creative Vitality.* Springfield, IL: Charles C. Thomas Publications.

[B25] Kaye S. Bleep M. (1997). *Arts and Healthcare.* London: Jessica Kinsley.

[B26] Kwak M. Zebrack B. J. Meeske K. A. Embry L. Aguilar C. Block R. (2013). Prevalence and predictors of post-traumatic stress symptoms in adolescent and young adult cancer survivors: a 1-year follow-up study. *Psycho Oncology* 22 1798–1806. 10.1002/pon.3217 23135830

[B27] Liebman M. (2003). *Art Therapy With Groups.* London: Jessica Kingsley.

[B28] Luutonen S. Sohlman B. Salokangas R. K. R. Lehtinen V. Dowrick C. (2011). Weak sense of coherence predicts depression: 1-year and 9-year follow-ups of the Finnish Outcomes of Depression International Network (ODIN) sample. *J. Mental Health* 20 43–51. 10.3109/09638237.2010.537401 21271825

[B29] Malchiodi K. (1999). *Medical Art Therapy with Adults.* London: Jessica Kinsley.

[B30] Minar V. (1999). *Art Therapy and Cancer: Images of the Hunter and the Healer.* London: Jessica Kingsley.

[B31] Mohanty C. T. (2003). “Under Western eyes: Feminist scholarship and colonial discourses,” in *Feminist Postcolonial Theory: A Reader*, eds Lewis R. Mills S. (Edinburgh: Edinburgh University Press).

[B32] Moksnes U. K. Espnes G. A. Lillefjell M. (2012). Sense of coherence and emotional health in adolescents. *J. Adolesc.* 35 433–441. 10.1016/j.adolescence.2011.07.013 21831417

[B33] Moksnes U. K. Rannestad T. Byrne D. B. Espnes G. A. (2011). The association between stress, sense of coherence and subjective health complaints in adolescents: sense of coherence as a potential moderator. *Stress Health* 27 157–165. 10.1002/smi.1353

[B34] Morse J. M. (1995). *Qualitative Research Methods for Health Professionals.* London: Chapman & Hall.

[B35] Nelson K. Fivush R. (2004). The emergence of autobiographical memory: a social cultural developmental theory. *Psychol. Rev.* 111 486–511. 10.1037/0033-295X.111.2.486 15065919

[B36] O’Connor M. Christensen S. Jensen A. B. Moller S. Zachariae R. (2011). How traumatic is breast cancer? Post-traumatic stress symptoms (PTSS) and risk factors for severe PTSS at 3 and 15 months after surgery in a nationwide cohort of Danish women treated for primary breast cancer. *Br. J. Cancer* 104 419–426. 10.1038/sj.bjc.6606073 21224851 PMC3049569

[B37] Pérez S. Galdón M. Andreu Y. Ibáñez E. Durá E. Conchado Peiró A. (2014). Posttraumatic stress symptoms in breast cancer patients: temporal evolution, predictors, and mediation. *J. Trauma Stress* 27 224–231. 10.1002/jts.21901 24659562

[B38] Riley S. Malchiodi C. (1994). *Integrative Approaches to Family Art Psychotherapy.* Chicago: Magnolia Street Publications.

[B39] Rubin J. (2001). *Approaches to Art Therapy.* Philadelphia, PA: Brunner and Maze.

[B40] Shapiro L. (ed.) (2014). *The Routledge Handbook of Embodied Cognition.* London: Routledge.

[B41] Wilkinson S. Kitzinger C. (2000). Thinking differently about thinking positive: a discursive approach to cancer patients. *Soc. Sci. Med.* 50 797–811. 10.1016/S0277-9536(99)00337-8 10695978

[B42] Wood M. J. M. Molassiotis A. Payne S. (2011). What research evidence is there the use of art therapy in the management of symptoms in adults with cancer? A systematic review. *Psychooncology* 20 135–145. 10.1002/pon.1722 20878827

[B43] Zelizer C. (2003). The role of artistic processes in peace-building in Bosnia-Herzegovina. *Peace Conf. Stud.* 10 62–75.

